# Effect of sitagliptin on tissue characteristics of the carotid wall in patients with type 2 diabetes: a post hoc sub-analysis of the sitagliptin preventive study of intima-media thickness evaluation (SPIKE)

**DOI:** 10.1186/s12933-018-0666-3

**Published:** 2018-02-05

**Authors:** Naoto Katakami, Tomoya Mita, Yoko Irie, Mitsuyoshi Takahara, Taka-aki Matsuoka, Masahiko Gosho, Hirotaka Watada, Iichiro Shimomura, on behalf of the Sitagliptin Preventive study of Intima-media thickness Evaluation (SPIKE) Collaborators

**Affiliations:** 1 Department of Metabolic Medicine, Osaka University Graduate School of Medicine, 2-2, Yamadaoka, Suita, Osaka 565-0871 Japan; 2 Department of Metabolism and Atherosclerosis, Osaka University Graduate School of Medicine, 2-2, Yamadaoka, Suita, Osaka 565-0871 Japan; 3 Department of Medicine, Metabolism & Endocrinology, Juntendo University Graduate School of Medicine, Hongo 2-1-1, Bunkyo-ku, Tokyo, 113-8421 Japan; 4 Center for Molecular Diabetology, Juntendo University Graduate School of Medicine, Hongo 2-1-1, Bunkyo-ku, Tokyo, 113-8421 Japan; 5 Osaka Police Hospital, 10-31 Kitayamacho, Tennoji-ku, Osaka, 543-0035 Japan; 6 Department of Diabetes Care Medicine, Osaka University Graduate School of Medicine, 2-2, Yamadaoka, Suita, Osaka 565-0871 Japan; 7 Department of Biostatistics, Faculty of Medicine, University of Tsukuba, 1-1-1, Tennodai, Tsukuba, Ibaraki 305-8575 Japan; 8 Center for Therapeutic Innovations in Diabetes, Juntendo University Graduate School of Medicine, Hongo 2-1-1, Bunkyo-ku, Tokyo, 113-8421 Japan

**Keywords:** DPP-4 inhibitor, Sitagliptin, Carotid atherosclerosis, Carotid plaque, Ultrasound diagnosis, Tissue characterization, Diabetes mellitus

## Abstract

**Background:**

Ultrasonic gray-scale median (GSM) of the carotid wall reflects its composition and low-GSM carotid plaque is considered to be vulnerable. This study aimed to evaluate the effect of sitagliptin, a dipeptidyl peptidase-4 inhibitor, on the longitudinal change in GSM, an index of the tissue characteristics of the carotid wall, in patients with type 2 diabetes mellitus (T2DM).

**Methods:**

This is a post hoc sub-analysis using data obtained from the SPIKE trial, a randomized controlled trial that demonstrated the beneficial effect of sitagliptin on the progression of carotid intima-media thickness in patients with T2DM. A total of 274 T2DM patients with no past history of apparent cardiovascular disease (137 in the sitagliptin treatment group and 137 in the conventional treatment group) were enrolled. The primary outcome was the change from baseline in mean GSM-CCA during the 104-week treatment period.

**Results:**

The mean GSM-CCA significantly increased in the sitagliptin treatment group (adjusted ΔGSM = 2.40 ± 1.19 [mean ± SE], p = 0.044) but not in the conventional treatment group (adjusted ΔGSM = 1.32 ± 1.19, p = 0.27). However, there was no significant difference in changes in mean GSM-CCA between the treatment groups.

**Conclusions:**

A post hoc sub-analysis suggests that the tissue characteristics of the carotid arterial wall were improved in the sitagliptin treatment group during the 104-week treatment period, but not in the conventional treatment group. However, there was no between-group difference in the changes of GSM values between the two treatment groups. Prespecified studies with large sample sizes would be necessary to confirm our findings.

*Trial registration* UMIN000028664, Registered 15 August 2017 (“retrospectively registered”)

## Introduction

Disruption of unstable atherosclerotic plaque plays a crucial role in the pathogenesis of cardiovascular disease (CVD) events. Plaque disruption is dependent on the tissue characteristics of the plaque lesion: the lipid content, the presence of neovascular vessels, inflammatory cells in the atheroma, and the thickness of the fibrous cap [1–4]. Since diabetes mellitus (DM) is related to increased vulnerability to plaque disruption and higher incidence of clinical CVD [5], stabilization of unstable plaque would be critical to reducing the incidence of CVD events in the management of DM.

Dipeptidyl peptidase-4 (DPP-4) inhibitors, which inhibit the degradation of active incretins including glucagon-like polypeptide-1 (GLP-1) by DPP-4, increase the concentration of active incretins and thereby improved glycemic control with low hypoglycemia risk. Furthermore, these agents potentially have anti-atherosclerotic properties beyond their glucoregulatory effects [6, 7]. Several experimental studies have shown that DPP-4 inhibitors can inhibit foam cell formation and atherosclerosis in both GLP-1-dependent and GLP-1-independent manners [8–13]. Such anti-inflammatory and anti-atherosclerotic effects of DPP-4 inhibitors have also been confirmed by studies conducted in clinical settings [14, 15]. Furthermore, several studies demonstrated that DPP-4 inhibitors prevented increase in carotid intima-media thickness (IMT), an established marker of early-stage atherosclerosis [16–19], in patients with type 2 diabetes (T2DM) compared with conventional treatment [20–22]. However, little is known about the effect of DPP-4 inhibitors on the tissue characteristics of atherosclerotic lesions in the carotid wall.

Interestingly, a recent study indicates that noninvasive ultrasonic tissue characterization of carotid plaque using gray-scale median (GSM) reflects plaque composition. Low-GSM plaque, which is characterized by high lipid content, neovascular vessels, and inflammatory infiltration, is considered to be particularly unstable [23]. Furthermore, the GSM value in the carotid wall can serve as a predictor of future CVD events [24–26]. The presence of echolucent low GSM plaque as well as plaque thickness were independent predictors of CVD, even after adjustment for other risk factors, and the addition of plaque echogenicity (presence/absence of echolucent plaque) and plaque thickness to the conventional risk factors and plaque thickness resulted in further and significant improvement of the risk prediction of CVD in asymptomatic patients with T2DM [25]. Thus, IMT and GSM reflect different aspects of atherosclerotic changes in the prediction of and the risk of cardiovascular disease. Therefore, it would be worthwhile to evaluate the effect of anti-diabetic agents on the longitudinal change in the GSM value.

The aim of the present study is to evaluate the effect of sitagliptin, a DPP-4 inhibitor, on the longitudinal change in the GSM value, an index of the ultrasonic tissue characteristics of the carotid wall, in patients with T2DM, using data obtained from the Sitagliptin Preventive study of Intima-media thickness Evaluation (SPIKE) trial [22].

## Research design and methods

The present study was a post hoc analysis based on data obtained from the Sitagliptin Preventive study of Intima-media thickness Evaluation (SPIKE) trial [22]. The original SPIKE trial was a prospective, randomized, open-label, multicenter, blinded end point (PROBE) study to evaluate the efficacy of sitagliptin in preventing the progression of atherosclerosis in patients with T2DM and the primary outcome was the change in carotid IMT during the 2-year intervention period. The study design, study schedule, and outcomes of the original SPIKE trial have been described in detail previously [22].

## Subjects

Participants eligible for the SPIKE trial were patients with T2DM in whom the target of blood glucose control specified in the Treatment Guide for Diabetes (Edited by Japan Diabetes Society in 2010) was not achieved (HbA1c ≥ 7.0%), despite insulin therapy in addition to dietary/exercise therapy or concomitant therapeutic drugs for T2DM other than DPP-4 inhibitors over a period of 3 months or longer. Exclusion criteria were (1) type 1 diabetes mellitus or secondary diabetes, (2) severe infections before or after surgery or severe trauma, (3) myocardial infarction, angina pectoris, cerebral stroke, or cerebral infarction, (4) retinopathy requiring laser photocoagulation and/or vitrectomy, or history of these treatments within 1 year, (5) moderate or severe renal dysfunction (serum creatinine: male, ≥ 1.4 mg/dL; female, ≥ 1.2 mg/dL), (6) severe liver dysfunction (aspartate aminotransferase ≥ 100 IU/L), (7) moderate or severe heart failure (New York Heart Association stage III or higher), (8) under treatment with an incretin preparation, such as other DPP-4 inhibitors, at the start of the study, (9) receiving therapeutic drugs not concomitantly administrable with incretin preparations with regard to the National Health Insurance program, such as DPP-4 inhibitors, at the start of the study, (10) pregnant, lactating, or possibly pregnant females or those planning to become pregnant, (11) medical history of hypersensitivity to investigational drugs, or (12) judged as ineligible by clinical investigators.

T2DM patients with no past history of apparent CVD who periodically attended the Outpatient Diabetes Clinics at 12 centers across Japan were asked to participate in this study and all patients who agreed to participate were enrolled. Originally, a total of 282 patients were enrolled and randomly allocated into either the sitagliptin treatment group (n = 142) or the conventional treatment group (using drugs other than the DPP-4 inhibitor) (n = 140). After excluding 8 patients from analyses (they withdrew from the study and/or objected to the inclusion of their data in any analysis), 137 subjects in the sitagliptin treatment group and 137 in the conventional treatment group were included in the full analysis set.

The protocols of the original study and this sub-analysis were approved by the Institutional Review Board of each participating institution in compliance with the Declaration of Helsinki and current legal regulations in Japan. Written informed consent was obtained from all the participants after full explanation of the study.

This study has been registered on the University Hospital Medical Information Network Clinical Trials Registry, which is a non-profit organization in Japan and meets the requirements of the International Committee of Medical Journal Editors (UMIN000007396, UMIN000028664).

## Ultrasound examination

B-mode ultrasonography of the carotid artery was performed using an ultrasound machine with a high-frequency (> 7.5-MHz) linear transducer. According to the guidelines of the Japan Society of Ultrasonics [27], scanning of the extracranial common carotid artery (CCA), the carotid bulb, and the internal carotid artery in the neck was performed bilaterally in three different longitudinal projections as well as transverse projections. The IMT was measured as the distance between 2 parallel echogenic lines corresponding to the blood-intima and media-adventitia interface on the posterior wall of the artery. The measurements of mean IMT of the CCA (mean-IMT-CCA) were performed using automated digital edge-detection software (IntimaScope; MEDIA CROSS, Tokyo, Japan) [28]. The software system averaged 200 points of IMT values in the segment 2 cm proximal to the dilation of the carotid bulb. The measurements of maximum IMT of the CCA (max-IMT-CCA) were performed at the site of the thickest point in the CCA. The method for determining IMT has been described in detail in previous reports [22].

The echogenicity of the arterial wall was evaluated based on the GSM method in a gray-scale range of 0–255 (0 as the darkest and 255 as the brightest tone). Adobe Photoshop software (Adobe Systems, version 7.0, San Jose, CA, USA) was used for image standardization and calculation of gray-scale values. In accordance with the previous report, the standardization of the B-mode image was performed using a curve option, so that the GSM for the blood ranged from 0 to 5, and for the adventitia from 185 to 195 [29]. Then, the mean-IMT-CCA area (the segment 2 cm proximal to the dilation of the carotid bulb) was delineated with a freehand tool, and the GSM of the selected area was read from the entire delineated area. Similarly, if there was an atherosclerotic plaque lesion (i.e. focal elevated lesion with max-IMT-CCA > 1.0 mm), the GSM of the plaque lesion was also measured using the same method. In the event multiple plaque lesions were found in one individual, the plaque with the greatest thickness was subject to GSM measurement and the GSM value was used as the subject’s representative value. To avoid inter-reader variability, all scans were electronically stored and read in random order by a single reader (K. A.) who was unaware of the clinical characteristics and the treatment group of the subjects.

## Outcome measures

The primary outcome of this study was the change of GSM value in the mean-IMT-CCA area (especially, the arithmetic average of the right and the left GSM values in one individual) during the 104-week observation period. In cases where atherosclerotic plaque lesions were found, the changes of the GSM value in the plaques were also evaluated. The definitions of the GSM measures used in this study are as follows:

- Mean GSM-CCA: the primary outcome of this study. The arithmetic average of the right and left GSM-CCA values. (If either the right or left GSM-CCA value was not obtained, the value of the other side was used as the mean GSM-CCA value).
- Right GSM-CCA: the GSM value of the intima-media complex measured in the mean-IMT-CCA area (the segment 2 cm proximal to the dilation of the carotid bulb) of the right common carotid artery.
- Left GSM-CCA: the GSM value of the intima-media complex measured in the mean-IMT-CCA area (the segment 2 cm proximal to the dilation of the carotid bulb) of the left common carotid artery.
- Right GSM-plaque: the GSM value of the plaque lesion (max-IMT-CCA > 1.0 mm) with the greatest thickness measured in the right common carotid artery.
- Left GSM-plaque: the GSM value of the plaque lesion (max-IMT-CCA > 1.0 mm) with the greatest thickness measured in the left common carotid artery.

## Statistical analyses

All values are reported as mean ± SD, median (range), or actual number of subjects with the percentage in parentheses.

The primary end point was the change in GSM of the carotid wall from baseline to 104 weeks. The primary analysis was performed using the mixed-effects model for repeated measures (MMRM) including treatment group, time (week), baseline GSM, and interaction between treatment group and time with an unstructured covariance structure to model within-subject variability. Subgroup analyses were also performed in subgroups by baseline sex, age, duration of diabetes, body mass index (BMI), HbA1c, presence of hypertension, presence of dyslipidemia, use of renin–angiotensin–aldosterone system inhibitors, and use of statins. In this subgroup analysis, differences in parameters from baseline to 104 weeks between groups were analyzed by the Student’s t-test. Baseline and follow-up group comparisons were assessed with the Student t test or Wilcoxon rank sum test for continuous variables and Fisher’s exact test for categorical variables.

To evaluate the associations between change in the mean GSM-CCA and other clinical parameters such as age, gender, body mass index (BMI), HbA1c, serum lipid levels (e.g. TC, HDL-C, TG), blood pressure, smoking status, administration of the anti-diabetic drugs other than insulin and DPP-4 inhibitors, anti-hyperlipidemic drugs, and anti-hypertensive drugs, and mean-IMT-CCA, regression analyses including treatment group as a covariate were performed.

All statistical tests were two-sided with a 5% significance level. Analyses were performed using SAS 9.4 software (SAS Institute Inc., Cary, NC).

## Results

### Baseline characteristics of study subjects

The baseline demographic and clinical characteristics of the 274 study participants (137 subjects in the sitagliptin group and 137 in the conventional treatment group) have been previously reported [22]. In summary, there were no significant differences between the sitagliptin group and the conventional treatment group in terms of the clinical parameters: the percentage of males was 61 and 60% (p = 1.00), age was 64 ± 10 and 64 ± 10 years (p = 0.90), the percentage of current smoker was 22 and 21% (p = 0.22), duration of T2DM was 17.2 ± 8.5 and 17.3 ± 8.7 years (p = 0.94), HbA1c was 8.1 ± 1.1 and 8.0 ± 1.0% (p = 0.45), BMI was 25 ± 4 and 25 ± 4 kg/m^2^ (p = 0.88), the prevalence of hypertension was 55 and 63% (p = 0.22), the prevalence of dyslipidemia was 66 and 61% (p = 0.45), and the percentage of statin use was 48 and 46% (p = 0.81), respectively..

### Effect of sitagliptin on metabolic factors and carotid IMT

Effect of sitagliptin on metabolic factors and carotid IMT have been also previously reported [22]. In summary, sitagliptin treatment had a more potent glucose-lowering effect than the conventional treatment (− 0.5 ± 1.0% vs. − 0.2 ± 0.9%, p = 0.004) without an increase in hypoglycemia. However, regarding serum lipid profiles, blood pressure, and markers of inflammation and endothelial injury (i.e. hs-CRP, interleukin-6, ICAM-1 and VCAM-1), there were no significant between-group differences in changes from baseline to 104 weeks (data not shown). Reductions in the mean and left maximum IMT, but not right maximum IMT, of the common carotid arteries at 104 weeks were significantly greater after sitagliptin treatment than after conventional treatment (− 0.029 mm [SE 0.013] vs. 0.024 mm [SE 0.013], p = 0.005; − 0.065 mm [SE 0.027] vs. 0.022 mm [SE 0.026], p = 0.021, and − 0.007 mm [SE 0.031] vs. 0.027 mm [SE 0.031], p = 0.45, respectively) [22].

### Effect of sitagliptin on the ultrasonic tissue characteristics of the carotid wall

At baseline, plaque lesions were observed in the right CCA in 139 subjects (74 subjects in the sitagliptin group and 65 in the conventional treatment group) and in the left CCA in 157 subjects (80 subjects in the sitagliptin group and 77 in the conventional treatment group), respectively. The GSM values of these plaques were measured. There were no significant differences in all the GSM measures (i.e. mean GSM-CCA, right GSM-CCA, left GSM-CCA, right GSM-Plaque, and left GSM-Plaque) between the two treatment groups at baseline (Table 1).

**Table 1:** The effect of sitagliptin on the GSM of the carotid wall

	n	Sitagliptin treatment group	n	Conventional treatment group	Adj. mean difference (95% CI)	p value between-group
Mean GSM-CCA (weeks)						
Baseline	*137*	*50.6* *±* *18.6*	*136*	*52.6* *±* *18.3*		*0.36*
52	*130*	*51.1* *±* *17.9*	*126*	*52.0* *±* *17.6*		*0.66*
104	*120*	*52.6* *±* *16.9*	*122*	*53.5* *±* *20.3*		*0.69*
Mean change (SE) (weeks)						
52		*0.65 (1.11)*		*0.25 (1.14)*	*0.40 (*− *2.74, 3.53)*	*0.80*
104		2.40 (1.19)*		*1.32 (1.19)*	*1.08 (*− *2.23, 4.39)*	*0.52*
Right GSM-CCA (weeks)						
Baseline	136	51.3 ± 20.6	136	52.8 ± 18.9		0.53
52	129	51.2 ± 19.1	126	53.1 ± 18.8		0.41
104	120	53.2 ± 18.2	122	53.4 ± 20.6		0.94
Mean change (SE) (weeks)						
52		0.14 (1.30)		1.26 (1.32)	− 1.13 (− 4.77, 2.53)	0.54
104		2.39 (1.38)		0.85 (1.37)	1.53 (− 2.30, 5.36)	0.43
Left GSM-CCA (weeks)						
Baseline	136	49.9 ± 19.9	136	52.5 ± 21.2		0.30
52	130	50.9 ± 19.4	126	50.9 ± 19.5		0.99
104	120	51.9 ± 19.0	122	53.6 ± 22.2		0.52
Mean change (SE) (weeks)						
52		1.12 (1.38)		− 0.46 (1.40)	1.58 (− 2.29, 5.45)	0.42
104		2.08 (1.48)		2.12 (1.48)	− 0.04 (− 4.17, 4.09)	0.98
Right GSM-plaque (weeks)						
Baseline	74	51.4 ± 19.5	65	55.8 ± 21.5		0.21
52	72	51.7 ± 19.3	56	55.6 ± 22.9		0.30
104	61	53.5 ± 20.4	62	59.2 ± 23.6		0.16
Mean change (SE) (weeks)						
52		2.06 (2.04)		2.80 (2.23)	− 0.74 (− 6.77, 5.28)	0.81
104		5.49 (2.69)*		4.20 (2.71)	1.29 (− 6.30, 8.88)	0.74
Left GSM-plaque (weeks)						
Baseline	80	52.6 ± 23.5	77	55.4 ± 20.9		0.43
52	76	51.2 ± 19.4	69	54.5 ± 20.5		0.32
104	73	54.5 ± 21.3	73	51.9 ± 17.5		0.42
Mean change (SE) (weeks)						
52		1.15 (2.19)		− 0.05 (2.24)	1.20 (− 5.01, 7.41)	0.70
104		5.10 (2.50)*		− 0.35 (2.57)	5.45 (− 1.66, 12.56)	0.13

The primary outcome of this study was the change of Mean GSM-CCA (sown in italics). Data are mean ± SD unless otherwise stated

Comparisons of GSMs during treatment with those at baseline were performed with one-sample t-test based on a mixed-effects model for repeated measures. Differences in Δchange in GSM from baseline at 52 and 104 weeks between groups were analyzed with a mixed-effects model for repeated measures. Treatment group, week, interactions between treatment group and week, and baseline GSM were included as fixed effects

*CI* confidence interval, *GSM* gray-scale median, *CCA* common carotid artery, *SE* standard error

* p < 0.05

The magnitude of the change in GSM values during the treatment period was evaluated using the MMRM (Table 1). The mean GSM-CCA significantly increased in the sitagliptin treatment group (adjusted ΔGSM = 2.40 ± 1.19 [mean ± SE], p = 0.044) but not in the in the conventional treatment group (adjusted ΔGSM = 1.32 ± 1.19, p = 0.27). In the sitagliptin treatment group, right GSM-Plaque (adjusted ΔGSM = 5.49 ± 2.69, p = 0.044) and left GSM-Plaque (adjusted ΔGSM = 5.10 ± 2.50, p = 0.044) also significantly increased during the 104-week observation period. Similarly, right GSM-CCA and left GSM-CCA tended to increase during the 104-week observation period, while it did not reach the statistical significance (adjusted ΔGSM = 2.39 ± 1.38, p = 0.084 and adjusted ΔGSM = 2.08 ± 1.48, p = 0.16, respectively). However, in the conventional treatment group, there were no significant changes in all the GSM measures (i.e. mean GSM-CCA, right GSM-CCA, left GSM-CCA, right GSM-Plaque, and left GSM-Plaque) during the 104-week observation period.

Similar findings were shown even after adjustment for possible confounding factors such as age, gender, BMI, HbA1c, serum lipid levels, blood pressure, smoking status, and administration of anti-diabetic, anti-hypertensive, anti-hyperlipidemic and anti-platelet drugs (data not shown).

However, there was no significant difference in the change in GSM measures from baseline at 52 and 104 weeks between the two groups.

**Fig. 1:**
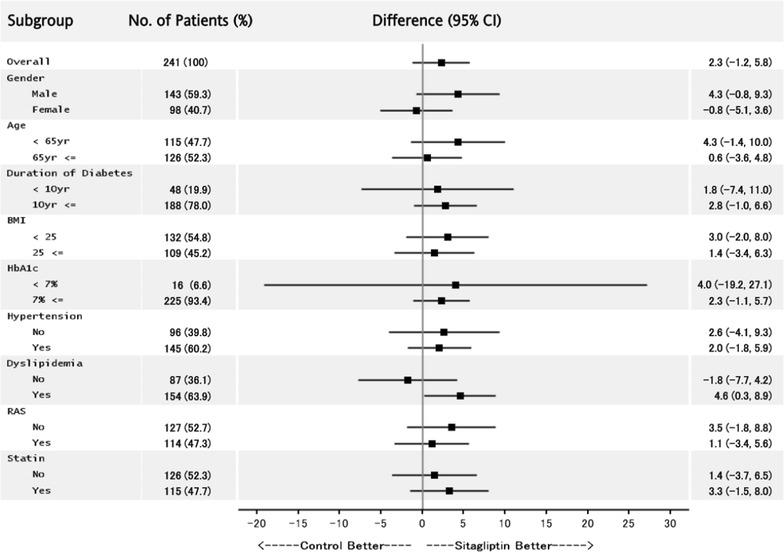
Differences in Δchange in mean GSM-CCA patients treated with or without sitagliptin in subgroup analysis. Differences in Δchange in mean GSM-CCA from baseline at 104 weeks in patients treated with or without sitagliptin were analyzed by the Student’s t-test. Subgroup analyses were performed in subgroups by baseline sex, age, duration of diabetes, BMI, HbA1c, presence of hypertension, presence of dyslipidemia, use of renin–angiotensin–aldosterone system inhibitors, and use of statins. Data are expressed as mean with 95% confidence interval. *CI* confidence interval, *BMI* body mass index, *RAS* inhibitors of renin–angiotensin–aldosterone system

Differences in Δchange in mean GSM-CCA in patients treated with or without sitagliptin in subgroups were shown as Fig. 1. This subgroup analysis revealed similar results, while there was a significant between-treatment-group difference in Δchange in mean GSM-CCA in patients with dyslipidemia.

Regression analyses revealed that there was no statistically significant association between change in the mean GSM-CCA and clinical parameters such as age, gender, BMI, HbA1c, serum lipid levels (e.g. TC, HDL-C, TG), blood pressure, smoking status, and administration of the anti-diabetic drugs other than insulin and DPP-4 inhibitors, anti-hyperlipidemic drugs, and anti-hypertensive drugs, and mean-IMT-CCA.

## Discussion

Many previous studies have provided the evidences that incretin-related agents such as GLP-1 analogues and DPP-4 inhibitors provide beneficial effects against atherosclerosis [7, 20–22, 30, 31]. Although the PROLOGUE trial, a study to evaluate whether DPP-4 inhibitors affect atherosclerosis, did not show an additional effect of sitagliptin on the progression of carotid IMT [32], several studies demonstrated that DPP-4 inhibitors more potently inhibited the progression of carotid IMT than conventional treatment in patients with T2DM [21, 22, 30]. However, it remains unclear whether these agents affect the tissue characteristics of the carotid arterial wall.

The present study, a post hoc sub-analysis using data obtained from the SPIKE study showed that sitagliptin treatment significantly increased the GSM value, an index of ultrasonic tissue characteristics, of the carotid arterial wall over a 104-week observation period, while conventional treatment did not affect the GSM value. This finding was complementary with the result of the original SPIKE study showing that sitagliptin treatment more potently inhibited the progression of carotid IMT than conventional treatment in patients with T2DM [22]. Our study was also consistent with another previous study using samples from asymptomatic patients undergoing carotid endarterectomy that revealed that incretin therapy–treated plaques presented with higher collagen content and less inflammation and oxidative stress than non-incretin–treated plaques, indicating a more stable plaque phenotype [33].

The precise mechanism how sitagliptin improved the tissue characteristic of the carotid arterial wall remains unclear. Hypercholesterolemia, oxidative stress, inflammation, and insulin resistance are major risk factors for the formation of the vulnerable plaque [34, 35]. However, in the present study, neither serum lipid profiles nor inflammation markers were significantly associated with change in the mean GSM-CCA. Improvement in hyperglycemia may be related to the improvement of the tissue characteristics of the carotid arterial wall, since reduction in HbA1c was tended to be larger in the sitagliptin treatment group [22]. Low plasma adiponectin levels are associated with increased plaque vulnerability [36]. Interestingly, atherosclerotic plaques of T2DM patients showed lower levels of adiponectin and adaptor protein PH domain and leucine zipper containing 1 (APPL1), an adaptor protein that interact directly with adiponectin receptors, compared with non-diabetic patients. It was also shown that current incretin-users presented higher adiponectin and APPL1 content compared with never incretin users [37]. These findings suggest a potential role of adiponectin/APPL1 signaling mediating the beneficial effect of sitagliptin on the tissue characteristic of the carotid arterial wall. In addition, recent studies suggest that DPP-4 may play a direct role in vascular inflammation and atherosclerosis independent of its metabolic actions [11, 38–40]. Thus, direct anti-atherosclerotic effect of sitagliptin on vascular cells is another possible explanation for its beneficial effect on carotid arterial wall, since DPP-4 inhibitors had anti-atherosclerotic effects in both a GLP-1-dependent and GLP-1-independent manners [8–13].

Several limitations of our study should be discussed. First, although a significant increase in GSM value during the treatment period was observed in sitagliptin treatment group alone, there was no between-group difference in the changes of GSM values between the two treatment groups. Therefore, it would be premature to conclude that sitagliptin treatment significantly improved the tissue characteristics of the carotid arterial wall. Second, the present study is a post hoc sub-analysis using data obtained from the SPIKE study. Third, the ultrasound settings for each image were not always standardized. However, the blood was used as the reference for black and the adventitia as the reference for white, and gain settings for measurements within an individual were similar throughout the study. Therefore, the impact of gain of ultrasound beam on the GSM value would be quite small, if any. Fourth, compared with the SPIKE trial showing beneficial effect of sitagliptin on progression of carotid IMT, in the PROLOGUE trial the change in carotid IMT in the sitagliptin group did not differ significantly from that in the conventional treatment group [22, 32]. As Oyama, et al. described in their paper, one possible explanation for this discrepancy is that higher prevalence of statin use in the PROLOGUE study as compared to the SPIKE trial masked the beneficial effect of sitagliptin, since statins potently suppress IMT progression. In addition, in the PROLOGUE study, additional use of both pioglitazone and biguanides increased in the conventional treatment group, possibly contributing to the suppression of IMT progression. Thus, administration of anti-diabetic, anti-hyperlipidemic, and anti-hypertensive drugs may affect the plaque components as well as its thickness. Although the baseline medical prescriptions were almost matched in this post hoc analysis of the SPIKE trial, it was not possible to adjust for the effect of changes in therapeutic regimen during the observation period. Another possible explanation for the different effect of sitagliptin on progression of carotid IMT between these two studies is that the baseline characteristics of the participants, such as severity of T2DM, HbA1c level, and the prevalence of hypertension, dyslipidemia, and cardiovascular disease, differed between them. Therefore, it would thus be premature to generalize our findings to the T2DM patients with higher risk for atherosclerosis.

## Conclusions

In conclusion, a post hoc sub-analysis suggests that the tissue characteristics of the carotid arterial wall were improved in the sitagliptin treatment group during the 104-week treatment period but not in the conventional treatment group. However, the statistically significant additional effect of sitagliptin on carotid GSM relative to conventional treatment was not demonstrated. Prespecified studies with large sample size would be necessary to confirm our findings.

## Abbreviations

BMI: body mass index
CCA: common carotid artery
CVD: cardiovascular disease
DM: diabetes mellitus
DPP-4: dipeptidyl peptidase-4
GLP-1: glucagon-like polypeptide-1
GSM: gray-scale median
HR: hazard ratio
IMT: intima-media thickness
MMRM: mixed-effects model for repeated measures
T2DM: type 2 diabetes mellitus
